# A high-throughput *in vivo* screening method in the mouse for identifying regulators of metastatic colonisation

**DOI:** 10.1038/nprot.2017.118

**Published:** 2017-11-02

**Authors:** Anneliese O. Speak, Agnieszka Swiatkowska, Natasha A. Karp, Mark J Arends, David J Adams, Louise van der Weyden

**Affiliations:** 1 Experimental Cancer Genetics, Wellcome Trust Sanger Institute, Wellcome Genome Campus, Hinxton, CB10 1SA; 2 Quantitative Biology, IMED, AstraZeneca, Darwin Building (Unit 310), Cambridge Science Park, Milton Road, Cambridge, CB4 0WG; 3 Cancer Research UK Edinburgh Centre, University of Edinburgh, IGMM, Crewe Road, Edinburgh, EH4 2XR

## Abstract

We describe a sensitive, robust, high-throughput method for quantifying the ability of metastatic tumour cells to colonise a secondary organ. Metastasis is the leading cause of death in cancer patients and successful colonisation of the secondary organ is the rate-limiting step in the metastatic process, thus experimental methods that can be used to interrogate the key factors required for this critical step are of great importance. The experimental metastasis assay we detail here includes tail vein injection of cancer cells into the mouse and determination of the resulting secondary organ colonisation, primarily in the lung, 10 days post-dosing. This assay can be used to investigate factors that regulate metastatic colonisation both at the tumour cell intrinsic level (via manipulation of the tumour cells prior to injection) and tumour cell extrinsic level (such as the tissue microenvironment, via the use of genetically modified mice or agents such as antibodies, drugs, etc). With this method we have robustly screened more than 950 genetically-modified mouse lines to identify novel microenvironmental regulators of metastatic colonisation. Experimental details discussed here include choosing appropriate cell numbers, handling of the cells, selection of recipient animals, and injection techniques. Further, we discuss key experimental design considerations, including the choice of method used to determine metastatic burden and statistical analysis of the results, as well as providing trouble shooting tips and identification of the factors that contribute to experimental variability.

## Introduction

**Figure 1:**
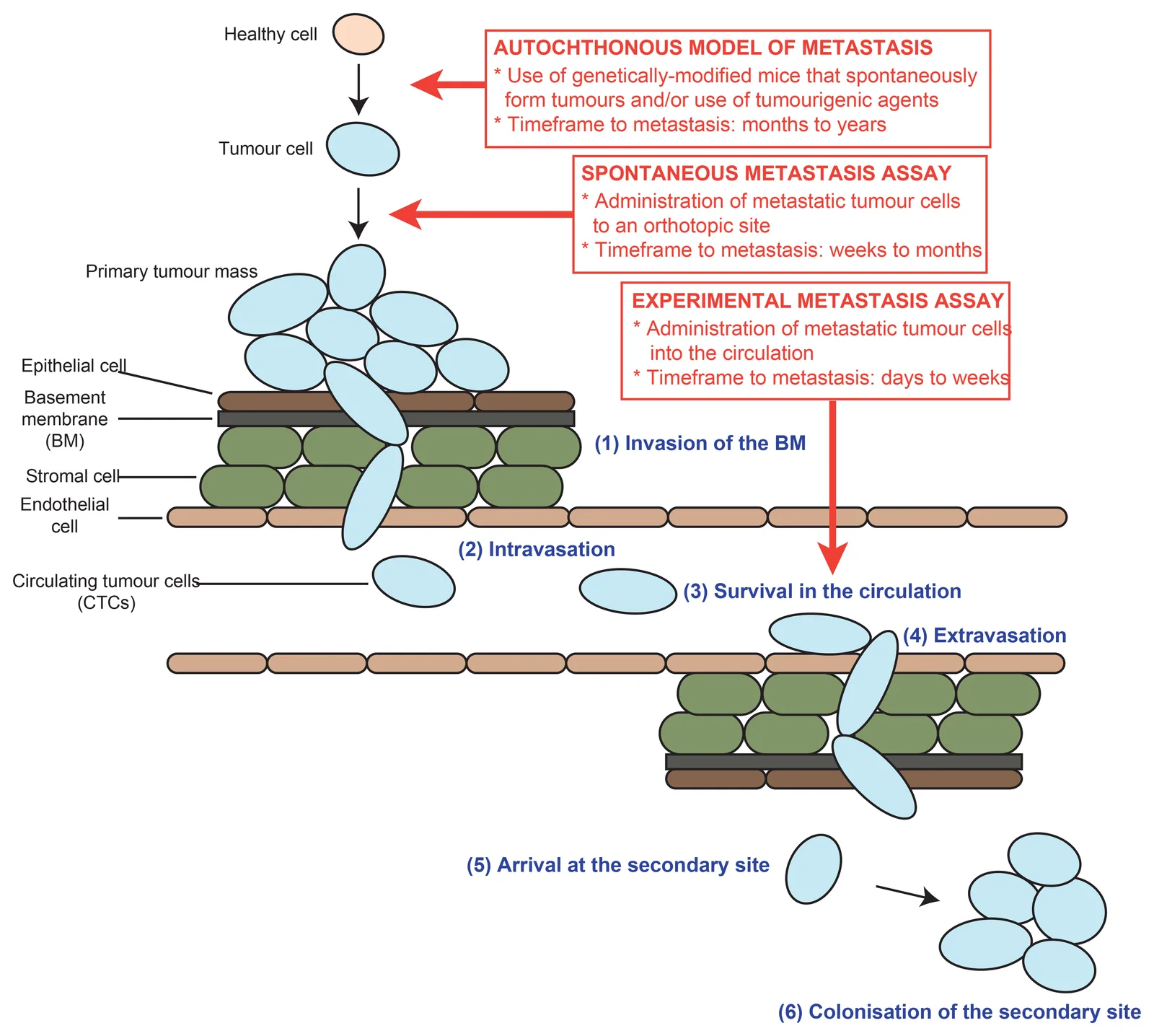
Schematic of the metastatic process and experimental procedures to model metastasis in the mouse. A healthy cell acquires the necessary mutations to become malignant and proliferates to form a primary tumour mass. (1) Metastasizing tumour cells invade the basement membrane, and (2) undergo intravasation into the circulation (or lymphatics). (3) The circulating tumour cells must then survive, arrest and (4) extravasate the circulatory system. (5) Survival after arriving at the secondary site is the rate-limiting step of the metastatic process, as the newly arrived tumour cell can undergo apoptosis, remain as a single cell or occult micrometastasis (which can remain dormant for years), or (6) proliferate and progressively colonise the organ. There are 3 ways to model metastasis in the mouse, involving the autochthonous model, the spontaneous metastasis assay or the experimental metastasis assay. The steps of the metastatic pathway mimicked by these techniques are indicated by the arrows, with the boxes detailing the nature and timeframe of each of these procedures.

Metastasis, a major contributor to cancer-related deaths, is a complex multi-step process, which involves the invasion of adjacent tissues, intravasation, transport through the circulatory system, arrest at a secondary site, extravasation and growth in a secondary organ (Figure 1). Tumour cells that have undergone intravasation are able to survive in the circulation with high efficiency and extravasate equally as efficiently, with >80% of cells successfully completing this process [reviewed in Chambers et al., 2000 ^1^], However the ability to extravasate *in vivo* is not necessarily predictive of subsequent metastasis formation (for example, metastatic ras-transformed and control fibroblasts extravasate equally well ^2^). Thus is it the post-extravasation steps that are much less efficient and more variable with *in vivo* videomicroscopy studies demonstrating that only a small proportion of extravasated cells begin to grow (~2% of injected cells), and only a subset of micrometastases continue to form macrometastases (~0.02% of injected cells) ^3^. Therefore, the ability to grow and ‘conquer’ a foreign environment, i.e., to ‘colonise’ a secondary site, is the rate-limiting step in the metastatic cascade.

The colonisation of a secondary organ is dependent upon both tumour cell intrinsic and extrinsic factors. The importance of tumour cell intrinsic factors is highlighted by exome sequencing of primary and matched metastatic tumour tissues from patients to identify the genetic divergence of metastases from the primary tumour ^4^, as well as the identification of metastasis-suppressor genes, such as *NM23* and *KISS1* [reviewed in Hurst & Welch, 2011 ^5^]. The importance of extrinsic factors, i.e., the microenvironment (or ‘host’), which includes fibroblasts, immune cells, and endothelial cells, is highlighted by reports identifying constitutional genetic polymorphisms that substantially influence the metastatic efficiency of tumours (such as *Sipa1* ^6^) and a recent study in which we used an experimental metastasis assay to screen 810 mutant mouse lines and identified 23 genes as host regulators of metastatic colonisation ^7^.

## Tools for studying metastatic colonisation

Much *in vitro* work has been performed to assess the metastatic propensity of tumour cells by examining their abilities in terms of adhesion and invasion. However, whilst these characteristics are necessary for metastasis, by themselves they are not sufficient as cells that lack the ability to complete other steps of the metastatic cascade, such as successful growth at the secondary site (‘colonisation’), may not form macroscopic metastatic lesions *in vivo* ^8–10^. In addition, *in vitro* assays do not take into account tumour cell extrinsic factors, such as the surrounding stroma, and the haematopoietic system. Thus the study of metastasis is ideally performed *in vivo*. The chorioallantoic membrane of the developing chick embryo is rich in blood vessels and tumour cell xenografts can be injected onto the membrane and their ability to intravasate and disseminate can be monitored ^11^. More recently, a high-throughput system for *in vivo* assessment of tumour cell intrinsic or microenvironmental modifiers of metastasis was described using zebrafish ^12^. However, the mouse remains the most widely used *in vivo* system for modeling metastasis, due to its physiological and genetic similarities to humans.

Modelling metastasis in the mouse can be achieved through the use of autochthonously arising metastatic lesions or transplantation models (Figure 1) [reviewed in Khanna and Hunter, 2005 ^13^]. Autochthonous (‘endogenous’) tumours capable of completing the entire metastatic process can be generated by the use of genetically-modified (GM) mice (such as *MMTV-PyMT* mice which develop mammary tumours that metastasize to the lung) and/or chemically-treated mice (such as 7,12-dimethylbenz(a)anthracene (DMBA)/12-O-tetradecanoyl-phorbol-13-acetate (TPA)-treated mice that form squamous cell carcinomas skin tumors that metastasize to the lymph nodes and lungs). However, it can take many months for the metastatic lesion to arise, and it can happen in a very asynchronous fashion (with great variability from mouse to mouse), so is not amenable to high-throughput screening. In addition, it does not allow modification of tumour cell intrinsic factors to determine how they affect the ability of the tumour cell to metastasize.

An alternative is the spontaneous metastasis assay, in which tumour cells are orthotopically transplanted into the mouse and allowed to invade the basement membrane and enter the bloodstream/lymphatics. An extension of this is “surgical orthotopic implantation” (SOI), in which histologically-intact fragments of human tumours are implanted to an orthotopic site [reviewed in Hoffman, 1999 ^14^]. Orthotopic implantation has the advantage that the tumour cells are placed in their ‘native’ microenvironment, which is likely to better reflect the activity of the tumour in patients, for example, Hoffman and colleagues showed that cisplatin had significant effects against small cell lung cancer tumours growing orthotopically, but was ineffective against the same tumours growing subcutaneously ^15^. In addition, the spontaneous metastasis assay allows interrogation of the early steps in the metastatic cascade, specifically, local invasion of the basement membrane and entrance into the vascular/lymphatic system. However, it can take weeks for metastasis to occur, and unless subcutaneously/intradermally injecting cells, this technique comes with the added complication that it involves surgery (such as administration of tumour cells into the mammary fat pads or brain) or use of anaesthetics and an imaging apparatus (such as administration of cancer cells directly into the colon, kidney or lung). In addition, if the primary tumour growth is rapid, surgery is often necessary to remove the tumour to both control morbidity and allow time for metastasis to occur (before the tumour reaches the size limit dictated by ethical committees) and removal of the primary tumour may affect the metastatic phenotype (primary tumour removal can both enhance and suppress metastasis, depending on the model studied).

An alternative is the experimental metastasis assay, in which cells are injected directly into the circulation. This can take as little as 10 days, does not require any imaging or surgical equipment or skills, and allows assessment of both tumour cell intrinsic and extrinsic factors of metastatic colonisation. We describe how to implement this assay here.

## Development and applications of the method

The experimental metastasis assay determines the colonisation ability/potential of tumour cells by the measurement of the tumour burden in specific organs of the animal after injection of the tumour cells directly into the haematogenous circulation. It has been a long-standing technique in the metastasis field, with studies in the early 1950’s demonstrating that the injection of rabbit V_2_ carcinoma cells into either the portal vein/hepatic artery or a systemic vein led to the development of tumors in the liver or lung, respectively ^16^. In support of the “seed and soil” concept that Stephen Paget hypothesized in 1889 to explain the non-random pattern of cancer metastasis ^17^, seminal work by Fidler and co-workers in the late 1970’s used the experimental metastasis assay to demonstrate that the organ selectivity of metastatic cells is dependent on both tumour cell properties and host factors ^18,19^.

**Figure 2:**
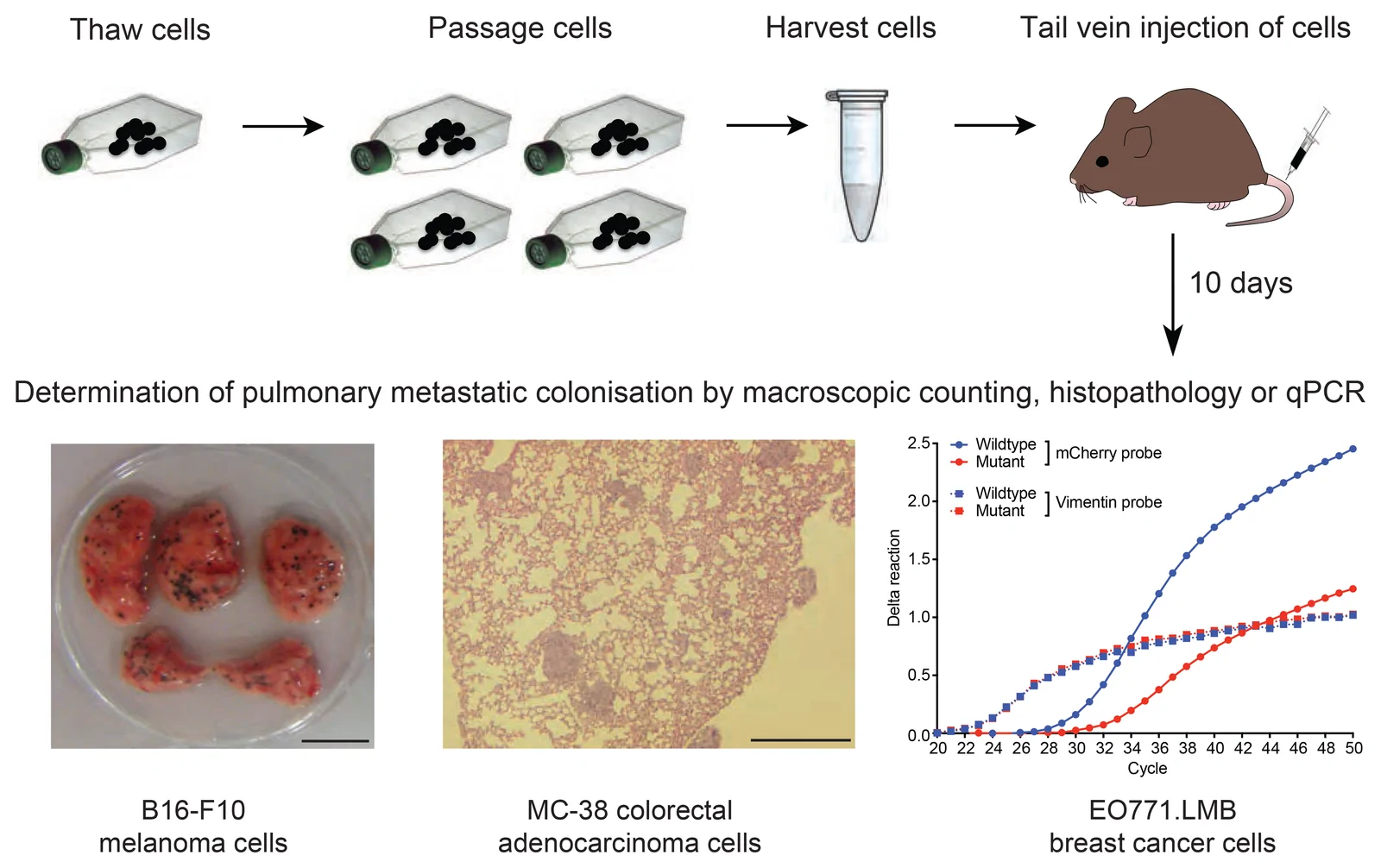
Schematic of the experimental metastasis assay using tail vein injection. The tumour cells are thawed and subsequently passaged before being harvested for tail vein injection into recipient mice. Ten days later, the tissue(s) can be collected (lungs are the predominant colonisation organ after tail vein administration) and examined for their metastatic tumour burden either by eye (if the cells are pigmented, such as the B16-F10 mouse melanoma cell line shown [4×10^5^ B16-F10 cells injected into a wildtype mouse; scale bar is 7 mm]), by light microscopy examination of haematoxylin and eosin-stained paraffin-embedded tissue sections (for all cell types, such as the MC-38 mouse colorectal adenocarcinoma cell line shown [5×10^5^ MC-38 cells injected into a wildtype mouse; scale bar is 0.5 mm]) or by quantitative polymerase chain reaction (qPCR) of the tissue for mCherry expression levels relative to endogenous vimentin expression levels (for all cell types once transfected with an mCherry-expressing plasmid, such as the EO771.LMB mouse breast cancer cell line [5×10^5^ EO771.LMB cells injected into a wildtype or homozygous *Arhgef1* mutant (*Arhgef1*^*tm1a(EUCOMM)Wtsi*^) mouse]). All data shown is representative of 2 independent experiments. Details on the cell lines, housing and husbandry of the mice used in these experiments and institutional regulatory board permission are given in Supplementary Methods.

Here we have taken the fundamentals of the experimental metastasis assay, and generated a method that is robust enough to be performed as a bespoke or high-throughput assay, to identify both tumour intrinsic and extrinsic factors that regulate metastatic colonisation. Our protocol involves intravenous injection of tumour cells into the lateral tail vein of mice, followed by determination of metastatic burden of the secondary organ, primarily the lung, 10 days later (Figure 2).

**Figure 3:**
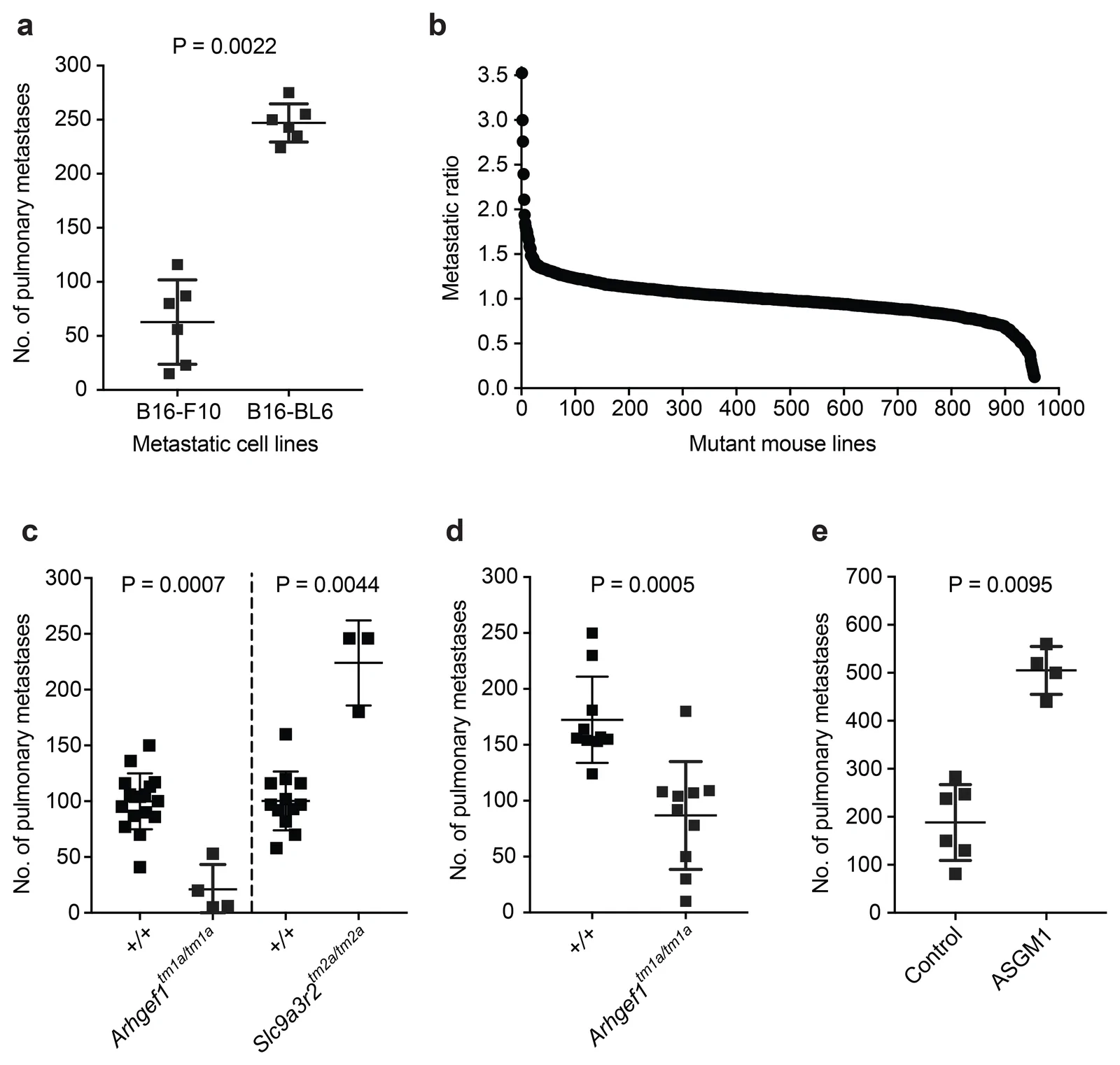
Applications for the experimental metastasis assay. **a**, Female mice were tail vein dosed with 1×10^5^ of either metastatic B16-F10 melanoma cells or highly metastatic B16-BL6 melanoma cells and the number of pulmonary metastatic foci counts at day 10. **b**, Mutant mouse lines (assayed with sex-, age-, and background matched wildtype controls) were tail vein dosed with B16-F10 cells and 10 days later the number of pulmonary metastatic foci counted by eye, with the result represented as a ‘metastatic ratio’ (mean number of metastases for the mutant line divided by the mean number of metastases for concurrently assayed wildtype controls, thus allowing comparison of mutant lines tested on separate days). **c**, Examples of mutant mouse lines, with respective controls (*+/+*), that show an ability to regulate metastasis with homozygous *Arhgef1* mutant (*Arhgef1*^*tm1a(EUCOMM)Wtsi*^) male mice showing decreased metastatic colonisation and homozygous *Slc9a3r2* mutant (*Slc9a3r2*^*tm2a(EUCOMM)Hmgu*^) male mice showing increased metastatic colonisation. **d**, Bone marrow chimaeras (irradiated female wildtype mice transplanted with donor bone marrow) were tail vein dosed with 4×10^5^ of B16-F10 melanoma cells and 10 days later the number of metastatic foci counted by eye. The donor bone marrow came from either wildtype (*+/+*) or homozygous *Arhgef1* mutant (*Arhgef1*^*tm1a(EUCOMM)Wtsi*^) mice. **e**, Wildtype female mice were *in vivo* depleted for natural killer (NK) cells by intra-peritoneal (i.p.) administration of ASGM1 antibody at -3 days, -1 hour and +5 days, with tail vein dosing of 4×10^5^ of B16-F10 melanoma cells at day 0, and counting of the number of metastatic foci at day 10. Squares represent individual mice with error bars shown as SD, and statistical analysis performed using Mann-Whitney test (for a, c, d, e). Circles represent individual mutant mouse lines (b). All data shown is representative of 2 (d, e) or 6 (a, c) independent experiments. Details on the cell lines, housing and husbandry of the mice used in these experiments and institutional regulatory board permission are given in Supplementary Methods. The data used to generate (b) and (d) was from a study that screened 810 mutant mouse lines for their ability to regulate metastatic colonisation ^7^.

Tumour cell intrinsic factors can be examined via prior manipulation of the tumour cell line *in vitro* (such as by CRISPR/Cas9). This could be done using both a targeted approach, in which guide RNAs are used to target a single gene to determine the metastatic capabilities of that modified cell line, or as a screening approach, in which a library of guide RNAs are used, to determine which gene(s) confers metastatic potential and enable successful colonisation of a secondary organ. This method can also be used to compare the metastatic potential of tumour cell lines that have undergone different selection processes *in vitro* or *in vivo* (Figure 3a). The cell lines can be either of murine origin (which allows injection into syngeneic wildtype or genetically modified mice and has the advantage of an immunocompetent host), or of human origin (if injected into immunodeficient mice, such as NOD-SCID mice, to avoid immunological rejection).

The role of tumour cell extrinsic factors can be examined by the use of genetically modified (GM) mice carrying specific genetic modifications, such as null alleles, point mutations, gain-of-function alleles or conditional alleles. Using this protocol we have screened 950 genetically-modified mouse lines to discover novel genes that contribute to microenvironmental regulation of metastatic colonisation (Figure 3b; the results of 810 mutant lines screened has been previously described ^7^). Examples of such genes include *Slc9a3r2* (*Slc9a3r2* mutant mice showed increased colonisation) and *Arhgef1* (*Arhgef1* mutant mice showed decreased colonisation; Figure 3c).

The use of bone marrow chimaeras, where the host animal is irradiated to eliminate the stem cells in their bone marrow so they can be replaced with donor bone marrow, allows examination of the role of the haematopoietic (immune) system in metastatic colonisation. For example, the decreased metastatic colonisation phenotype observed in *Arhgef1* mutant mice is mediated, at least in part, by the haematopoietic system, as bone marrow chimaeras given *Arhgef1* mutant bone marrow also showed a decreased metastatic colonisation phenotype (Figure 3d). Similarly, when used in combination with *in vivo* depletion techniques, in which animals are depleted of specific components of the haematopoietic system via administration of antibodies, the role of individual haematopoietic cell types in metastatic colonisation can be interrogated. For example, administration of anti-asialo ganglio-N-tetraosylceramide (ASGM1) antiserum to deplete natural killer (NK) cells, results in increased metastatic colonisation, confirming the key role of NK cells in controlling metastasis (Figure 3e). Finally, this method can also be used in a pre-clinical setting, to assess the effectiveness of potential therapeutic drugs on metastatic colonisation [reviewed in Steeg, 2016 ^20^].

## Limitations of the method

As the experimental metastasis assay involves injection of cancer cells directly into the bloodstream, it bypasses the early steps of the metastatic pathway, specifically the invasion of the cancer cells into the surrounding tissue and subsequent intravasation. To investigate these early steps of the metastatic cascade, use of the ‘spontaneous metastasis assay’ or ‘autocthonous metastasis’ would be a more appropriate technique. However, it can be argued that these early steps are not the ‘critical’ ones as many tumour cells break away from the primary tumour mass and can be found in the blood stream as ‘circulating tumour cells’ (CTCs). The ability to generate CTCs is however, not indicative of their metastatic potential as preclinical models have demonstrated that within 24 hours of intravenous administration, less than 0.1% of the tumour cells remain viable and that less than 0.01% of these surviving CTCs can produce metastases ^21^.

After tail vein injection, the first capillary bed that the tumour cells encounter are those of the lung (if they pass through the lungs, they enter the arterial system and eventually pass into the portal circulation) and thus the experimental metastasis assay using the tail vein route tends to favour colonisation of the lung. However, it can be argued that metastases in the lung are found in many cancer types (breast cancer, colon cancer, bladder cancer and melanoma, to name a few ^22^), so it is a highly clinically relevant organ for investigation of colonisation factors. Furthermore, although not commonly used, there are some tumour cell lines that have been shown to colonise other organs after tail vein injection; EL-4 cells (a C57BL/6 dimethylbenzanthracene-induced thymoma line ^23^), ESb cells (a DBA/2 methylcholanthrene-induced lymphoma line) ^24^ and RAW117-H10 cells (a BALB/c large-cell lymphoma line ^25^) all colonise the liver after tail vein injection. In contrast, other cell lines colonise multiple organs, such the C57BL/6J methylcholanthrene-induced pleomorphic myofibrosarcoma ER 15-P cell line, that colonises the lungs, mediastinal lymph nodes, liver, kidneys and brain (with repeated administration of tumor cells from liver metastases into the tail vein leading to selection of a tumor cell line with a tendency to liver metastasis) ^26^. Alternatively, injection of tumour cells into the portal vein or left cardiac ventricle leads to colonisation of the liver and bone/brain, respectively.

## Experimental design

### Mice and cells

**Figure 4:**
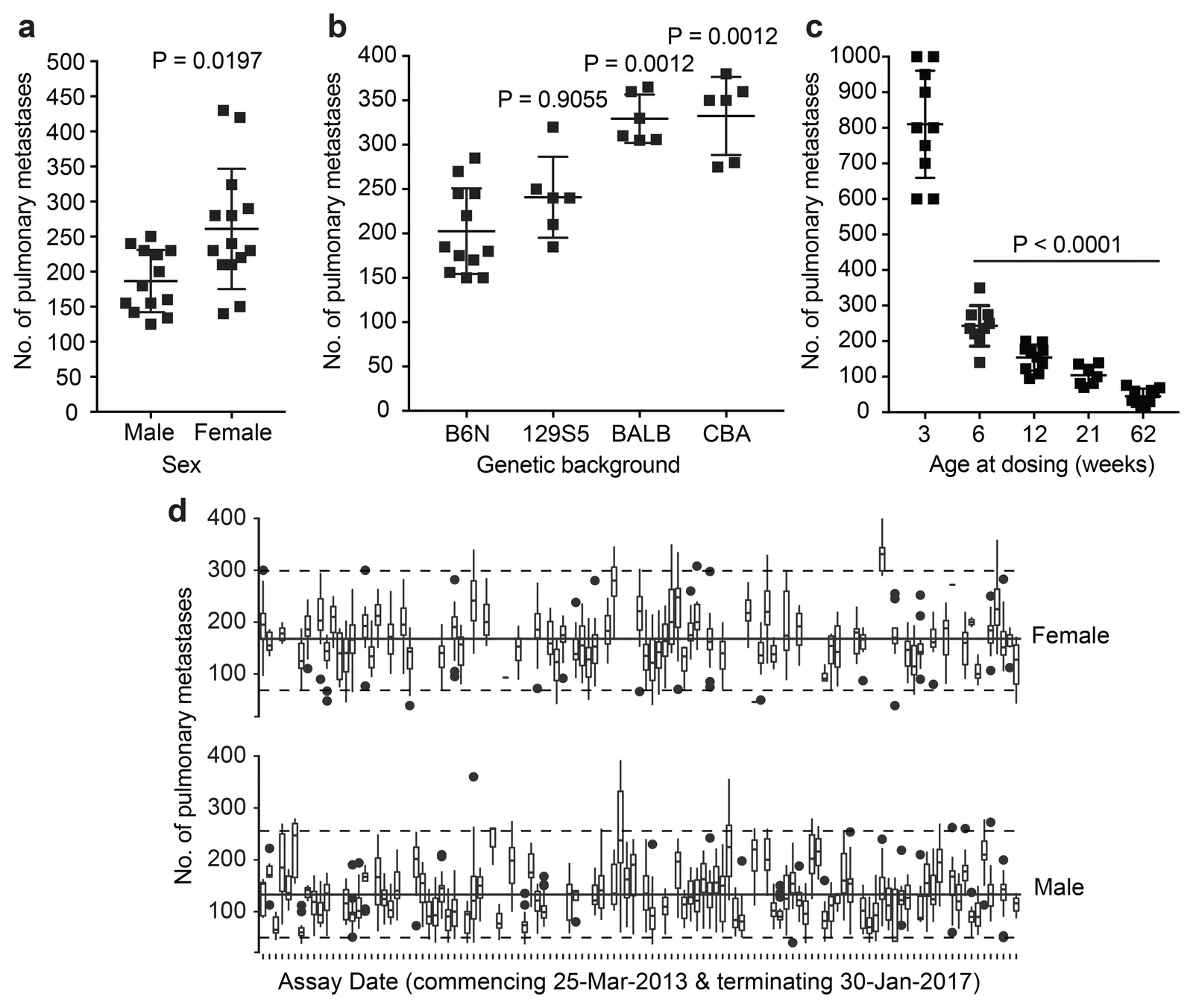
Effect of sex, genetic background and age on the experimental metastasis assay metastatic colonisation and variation of results of the assay over time. **a**, Wildtype male and female mice were tail vein dosed with 5×10^5^ B16-F10 cells. Squares represent individual mice with error bars shown as SD. Statistical analysis was performed using Mann-Whitney t test. **b**, Female mice tail vein dosed with 5×10^5^ B16-F10 cells, squares represent individual mice with error bars shown as SD. Statistical analysis was performed using a Kruskal-Wallis test with post hoc testing using a Dunn’s multiple comparisons test. Abbreviation of strains B6N = C57BL/6N, 129S5 = 129S5/SvEvTac, BALB = BALB/cOlaHsd and CBA = CBA. **c**, Female mice of different ages were tail vein dosed with 4×10^5^ B16-F10 cells. Squares represent individual mice with error bars shown as SD. Statistical analysis was performed using a Kruskal-Wallis test with post hoc testing of all groups against other using a Holm-Sidak’s multiple comparisons test (the P value shown is comparing the 3 week age with each of the other age groups). **d**, Graph showing the variation in the metastasis count over a 46-month period (within the dates shown) of wildtype mice on a B6N background, aged 7.5 to 9.4 weeks, administered 4×10^5^ B16-F10 cells. Vertical ticks indicate individual assay dates, solid horizontal line indicates the mean and dashed horizontal line indicates the 95% confidence interval. The boxplot represents a five point summary of minimum, first quartile, mean, third quartile and maximum, which excludes outliers if they are >1.5 times the interquartile range (Q3-Q1) from the relevant quartile, and these are shown as circles on that day. The data used to generate (d) was from a study that screened 810 mutant mouse lines for their ability to regulate metastatic colonisation ^7^. The data shown (a-c) is representative of 2 independent experiments. Details on the cell lines, housing and husbandry of the mice used in these experiments and institutional regulatory board permission are given in Supplementary Methods.

Mice should be matched by sex, age and genetic background as all of these factors can influence metastatic colonisation, as discussed below (**Box 1** and Figure 4). The experimenter should ensure that the experiment is performed in accordance with local animal research regulations. The tumour cell line should be thawed and passaged as minimally as possible prior to injection, to ensure cells maintain their potency *in vivo*. In this protocol we provide details for the B16-F10 mouse melanoma cell line that is widely used, dosing mice with 4×10^5^ cells in a 0.1mL volume, however, many other tumour cell lines can be used. When using a new tumour cell line, it is advisable to perform a pilot study with a range of doses (2-3 mice per dose), such as 0.5×10^5^, 1×10^5^, 5×10^5^ and 10×10^5^, to find which one generates sufficient colonies (ideally 50-100) to allow for modulations in metastatic colonisation to be detected (i.e., altering tumour cell intrinsic or extrinsic factors to result in increased or decreased metastatic colonisation).

The main sources of variation in the experimental metastasis assay that need to be considered in the experimental design are sex of the mice, genetic background of the mice, age of the mice and the assay date (Figure 4). We found that female mice had significantly higher rates of pulmonary metastatic colonisation (increased number of metastatic colonies on the lungs) compared to males (Figure 4a). Thus it is important to use sex-matched mice. The genetic background of the mouse can affect the number of pulmonary metastases (Figure 4b), which is most likely due to MHC (*H2*) haplotype differences between the strain of the mouse and the strain from which the tumour cell line was derived. For example, the B16-F10 mouse melanoma cell line is derived from a C57BL/6 mouse, and we observed significant differences pulmonary metastatic colonisation of these cells between C57BL/6 and 129 mouse strains (which are both *H2*^*b*^) and the BALB/c and CBA mouse strains, which are *H2*^*d*^ and *H2*^*k*^, respectively. Thus it is important to ensure that the control and mutant mice used in any experimental metastasis assay are strain-matched. We found that the age of the mouse can affect pulmonary metastatic colonisation ability, with younger mice having a higher tumour burden (Figure 4c). This may be a due to a relatively immature immune system and/or decreased blood volume in younger mice relative to older mice. Thus the age of the mouse is an important fact to consider and we recommend using mice at 6-8 weeks of age wherever possible.

By performing the experimental metastasis assay as part of a large-scale phenotyping pipeline, we found that similar to other phenotyping variables ^27^, assay date is a major source of variation (Figure 4d). This variation arises from multiple sources, including the researcher, culture and preparation of tumour cells and the cage. As this temporal variation has significant impact on data analysis, with counts done on the same day being more similar to themselves than counts done on another day, the solution is to compare mice assayed on different days using the ‘metastatic ratio’ (MR). The MR is calculated by dividing the counts for the experimental mice by the counts for the control mice dosed on the same day (this can be either mutant mice versus control mice (Figure 3b) or mice dosed with modified cells versus control cells).

### Statistical power

Performing power analysis to estimate the sample size required is an important aspect of experimental design. If sample size is too low, false positives can be common and the experiment can incorrectly conclude that no effect is present whilst if the sample size is too large, time and resources will be wasted. Statistical power for a test of statistical significance, depends on the size of the effect, the number of measures collected, the significance threshold used (typically fixed at 0.05), and the variability in the data. From the four factors that influence statistical power, the number of measures is the element the experimenter has control over. We can estimate statistical power for a variety of designs if we have a measure of variability and effect size of interest. To assess statistical power for the metastatic screen, we need an estimate of variability in the metastatic burden: using C57BL/6 wildtype data, we found that the average metastatic burden for mice dosed with 4×10^5^ B16-F10 cells was 139 with a standard deviation of 36 for males and 200 with a standard deviation of 45 for females (n=21/sex). The effect size needs to be a biological interest effect for the community, so within our program we implemented a metastatic ratio filter, which was equivalent to a 60% change in mean. Using these parameters, when 5 measures are collected per treatment, a power analysis for a two-tailed Mann-Whitney test, using G*Power 3.1.9.2, assuming the underlying distribution is normal, found we had a statistical power of 0.921 and 0.948 for males and females, respectively. Where statistical power (or sensitivity) is the probability of making the correct decision when the biological effect actually exists. From this data, to account for the natural variation in the metastatic count and detect a 60% change in mean the recommended design is 5 measures per group.

### Determination of metastatic burden

Wherever possible, the individual performing the assessment of metastatic burden should be blinded to the identity of the samples (e.g., which samples came from the mutant/drug-treated mice versus the control/vehicle-treated mice, or which samples were from mice dosed with modified cells versus mice dosed with unmodified cells). The metastatic burden of any tissue can be determined in four ways, depending upon the cell line administered: visually counting the number of lesions, counting of the number of lesions from H&E-stained sections of the tissue, *in vivo* imaging for bioluminescence or fluorescence detection or qPCR for detection of a marker.

**Figure 5:**
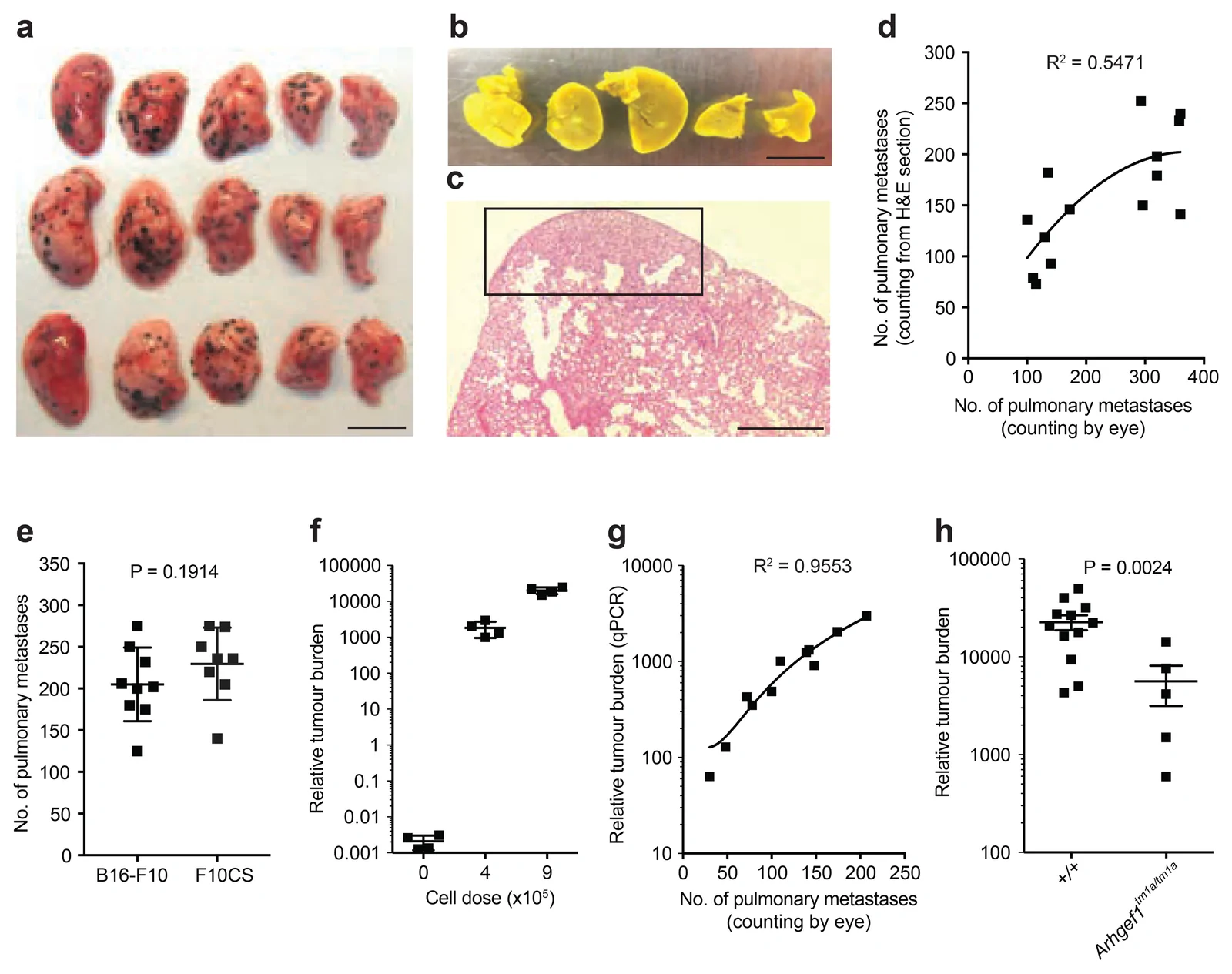
Methods for determining metastatic burden of a tissue. **a**, Representative macroscopic image of lungs from three individual wildtype female mice, tail vein dosed with 4×10^5^ B16-F10 mouse melanoma cells (the black spots on the pink lobes of the lung are the metastatic colonies that can be counted by eye). Scale bar is 7 mm. **b**, Representative image of Bouin’s stained lungs from a male mouse, tail vein dosed with 1×10^6^ mouse LL/2 lung carcinoma cells (the white spots on the yellow lobes of the lung are the metastatic colonies that can be counted by eye). Scale bar is 7 mm. **c**, Representative image of H&E-stained lungs from a wildtype male mouse, tail vein dosed with 1×10^6^ LL/2 mouse lung carcinoma cells (the box shows a large metastatic colony on a haematoxylin and eosin-stained, paraffin-embedded section of mouse lung). Scale bar is 0.5 mm. **d**, Comparison of two methods of determining pulmonary metastatic colonisation (counting from H&E-stained paraffin sections versus counting by eye) on female and male wildtype mice dosed with 4×10^5^ B16-F10 cells and the number of metastatic foci on the lungs of each mouse at day 10 first counted by eye, then put into formalin for histopathology processing (R^2^ value determined using a second order polynomial (quadratic) non-linear fit). **e**, Wildtype female mice tail vein dosed with 4×10^5^ of either B16-F10 mouse melanoma cells or F10CS cells (B16-F10 cells stably-expressing mCherry) and pulmonary metastatic foci counted at day 10, show no difference in metastatic potential between the two cell lines (i.e., stable transfection of mCherry has not altered the metastatic properties of the cells). Squares represent individual mice with error bars shown as SD, and statistical analysis performed using a Mann-Whitney test. **f**, Wildtype female mice tail vein dosed with different numbers of F10CS cells and ‘metastatic burden’ determined at day 10 by qPCR on DNA extracted from the lungs (expression level of mCherry relative to endogenous vimentin expression), demonstrate the sensitivity of the assay. **g**, Comparison of two methods of determining pulmonary metastatic colonisation (qPCR versus counting by eye) on female wildtype and mutant mice dosed with 4×10^5^ F10CS cells and the number of metastatic foci on the lungs of each mouse at day 10 first counted by eye, then DNA extracted from the lungs (R^2^ value determined using a second order polynomial (quadratic) non-linear fit). **h**, Female wildtype (*+/+*) and homozygous *Arhgef1* mutant (*Arhgef1*^*tm1a(EUCOMM)Wtsi*^) mice tail vein dosed with 4×10^5^ mCherry-expressing EO771.LMB cells and the lungs collected at day 10 for DNA extraction and qPCR (mCherry/vimentin). Squares represent individual mice with error bars shown as SEM, and statistical analysis performed using an unpaired two-tailed t test with Welch’s correction. The data shown (a-c, e-f, h) is representative of 2 independent experiments. Details on the cell lines, housing and husbandry of the mice used in these experiments and institutional regulatory board permission are given in Supplementary Methods.

Macroscopic counting of the number of metastatic lesions by eye (or under a low-powered/dissecting microscope) immediately after dissection has the advantage that it is quick, easy and no specialized skills or equipment are required (Figure 5a). The disadvantage of this, however, is that it is only applicable to pigmented cell lines, such as melanomas. Alternatively, it is possible to fix organs in Bouin's solution for 24 hours, followed by 100% ethanol washes, which makes the tumour cells appear white, and thus able to be counted (Figure 5b). Fixation of the relevant tissue, followed by embedding in paraffin, sectioning and haematoxylin and eosin (H&E) staining is a method that can be used to determine metastatic colonisation of any organ, by any tumour cell line administered (Figure 5c-d). This technique has the advantage that the tissue can be serially sectioned and thus the tumour burden of the whole tissue/organ can be determined. It can also allow for measurement of the individual metastatic lesions, thus can provide both quantitative and qualitative information about the metastatic colonisation phenotype of that mouse. It can also inform as to the presence of other cell types present in the organ, such as immune infiltrates. The disadvantage is that it depends on the researchers access to histology tools/services and/or a pathologist, and is not as quick as the macroscopic method.

The metastatic burden of a tissue can be determined by *in vivo* imaging once the tumour cell line has been transfected with a bioluminescence- (luciferase) or fluorescence-expressing construct. The advantage of this is that the mouse can be repeatedly imaged so that the developing metastases can be observed over a time period. The disadvantages are that the mouse needs to be dosed with luciferin prior to each imaging session (if bioluminescence is used) and ideally shaved to remove fur (unless a nude mouse is used). It can also be difficult to visualise the metastases if the metastatic burden is low ^28^ and requires access to expensive specialist imaging equipment (such as the IVIS system (Caliper LifeSciences) or Pearl Trilogy (Li-Cor)) ^29^. Alternatively, fluorescently tagged tumour cells, such as those expressing green fluorescent protein (GFP) or red fluorescent protein (RFP), can be detected using fluorescent imaging cameras (such as the OV100 imaging system (Olympus)) either independently or together with ultrasound imaging ^30^. Whilst the OV100 allows real-time imaging in live animals, a limitation is that the working distance (objective to animal) is approximately 4 cm so it may be necessary to perform surgery to access the organ of interest ^31^. Once a cell line has been transfected with a bioluminescent/fluorescent marker, quantitative polymerase chain reaction (qPCR) can also be used to determine metastatic colonisation (Figure 5e-h). In this case, the tissue can be collected for DNA extraction and qPCR performed to assess the level of the marker (from the tumour cells) relative to the level of vimentin (from both the tumour cells and the tissue; an internal control). The advantage of this technique is that the total tumour burden of the tissue can be assessed, rather than only what appears on the surface of the tissue (macroscopic method) or on the particular section (histopathology method). The disadvantage of this technique is that it requires transfection/drug selection of the cell line, which may be difficult depending on its suitability to be transfected and may also alter the *in vivo* properties of the cell line. This method also requires access to a qPCR machine.

### Statistical analysis

To avoid temporal effects confounding the statistical analysis, test and control samples should always be run concurrently. If a low sample size within a single experimental run, we recommend the use of the non-parametric Mann-Whitney to identify significant differences between the groups before assessing the biological significance using the ‘metastatic ratio’. We suggest both a statistical significance test and biological effect size cut off to select a treatment for further screening (for instance in our screen we used a Mann-Whitney P value of ≤ 0.0175 with a metastatic ratio of ≤ 0.6 or ≥ 1.6 ^7^). If significant in the initial cohort assayed, the experiment should be independently repeated an additional three times and the data from all cohorts processed through an integrative data analysis to determine those lines that were statistically significant. This two-step process ensures robust reproducible findings are identified.

Integrative Data Analysis ^32^ assesses for treatment effect across multiple experiments by treating each experiment as a fixed effect. We recommend implementing an iterative top down modeling strategy, starting with the most comprehensive model (either Eq. [1], or [2]) appropriate for the collection strategy implemented and ensuring the model only includes terms where the terms can be independently assessed [full details including code is published elsewhere: van der Weyden et al., manuscript submitted].

[Eq.1] $$\text{Y}={\text{β}}_{0}+{\text{β}}_{1}\;\text{Sex}\;+{\text{β}}_{2}\;\text{Experiment}\;+{\text{β}}_{3}\;\text{Treatment}\;+{\text{β}}_{4}\;\text{Sex}*\text{Treatment}\;$$

[Eq.2] $${\text{Y=β}}_{0}+{\text{β}}_{2}\text{Experiment}+{\text{β}}_{3}\text{Experiment}$$

The optimisation process first selects a covariance structure for the residual, then the model reduced by removing non-significant fixed effects, and finally the treatment effect is tested and model diagnostics visualised. The issue of multiple testing can be managed by adjusting the calculated p values to either control the family wise error rate (FWER) or the more sensitive false discovery rate (FDR).

## Materials

### Reagents

- Cells (typically established tumour cell lines, of human or mouse origin). **! CAUTION** Ensure the cells have been tested and found to be free of mycoplasma and any mouse pathogens. Also, when using cell lines, ensure these are authenticated.
- Cell culture medium (with serum and supplements) and reagents for culturing the cells (depending on the specific requirements of the cells).
- Recipient mice (age-, sex- and strain-matched adult mice, ideally 6-8 weeks of age). **! CAUTION** All animal studies should be carried out according to all relevant governmental and institutional animal care and research legislation and regulations. **★ CRITICAL STEP** Choice of mouse strain will be dependent on the cells being used and is discussed in the Introduction.
- Wet-ice.
- Dulbecco’s phosphate buffered saline (without Ca^2+^ and Mg^2+^) [PBS, Sigma, D8537].
- 1 mL syringes [BD Plastipak, 300013].
- 27G, 13mm needle [Becton Dickinson, 300635].
- Safelock 2mL microcentrifuge tubes [Eppendorf, 0030 120.094].
- 50 mL conical tube [Falcon, 352098] or 15 mL conical tube [Falcon, 352097].
- *Optional* (if performing analysis of metastatic burden by qPCR):
  ♦ TBSTx: 10x Tris Buffered Saline [Sigma, T5912] diluted to 1x Tris Buffered Saline (with ultra-pure water) and Triton X-100 [Sigma, T8787]. **! CAUTION** Harmful if swallowed, and causes skin irritation and eye damage so wear gloves, safety glasses and a laboratory coat.
  ♦ reagents to extract genomic DNA from tissue homogenates (we use Gentra Puregene Cell Kit [Qiagen, 158767]). **! CAUTION** Requires the use of proteinase K [Sigma, D8044]; if purchased in powder form, it may cause skin and respiratory irritation so needs to be handled in a chemical safety hood with gloves and a laboratory coat.
  ♦ qPCR kit for probe based assays (we use QuantiNova probe PCR kit [Qiagen, 208354]). **! CAUTION** Suspected teratogen so wear gloves, safety glasses and a laboratory coat.
  ♦ qPCR consumables (96 well plates, eg. MicroAmp Optical 96-well reaction plate [Life Technologies, N801-0560] and seals [Optical Adhesive Covers [Life Technologies, 4360954]).
  ♦ Vimentin (VIC conjugated primer limited) and mCherry (FAM conjugated) from Life Technologies (as shown below): **! CAUTION** Suspected carcinogen and teratogen wear gloves, safety glasses and a laboratory coat.
    Mouse vimentin (request primer limited format) - primer and probe sequences AGC TGC TAA CTA CCA GGA CAC TAT TG
    CGA AGG TGA CGA GCC ATC TC
    VIC - CCT TCA TGT TTT GGA TCT CAT CCT GCA GG – TAMRA
    mCherry-primer and probe sequences
    GAC CAC CTA CAA GGC CAA GAA G
    AGG TGA TGT CCA ACT TGA TGT TGA
    6FAM - CAG CTG CCC GGC GCC TAC A - TAMRA

### Equipment

- Cell culture incubator (humidified, and supplied with CO_2_, depending on the requirements of the cell line).
- Class II laminar flow hood for cell culture work.
- Pipette aid with sterile pipettes.
- Centrifuge with swing-bucket rotor.
- Pipette and pipette tips.
- Cell counter (electronic or haematocytometer).
- PPE for handling mice (as required by institutional regulations).
- Heated rodent chamber.
- Rodent restrainer.
- Necropsy equipment (scissors and tweezers, exact type depends upon personal preference).
- *Optional*: light microscope (if determining metastatic burden by H&E-stained paraffin sections) or method to homogenise tissues (we use a gentleMACS**™** Octo Dissociator with gentleMACS**™** M Tubes [Miltenyi Biotec; 130-095-937 and 130-096-335, respectively] and qPCR instrument and analysis software (if determining metastatic burden by mCherry qPCR).

## Procedure

### Passaging of cells to be injected • TIMING 30 min (2-3 d before animal injection)

1. Use standard procedures recommended for the specific cell line in use and passage the cells such that on the day of the experiment the cells are ~80% confluent. Ideally, the cells should be passaged no sooner than 2-3 days prior to the experiment, to ensure they are in log-phase growth.
  **★ CRITICAL STEP** The cells should be passaged at least once after being removed from liquid nitrogen before being used in the assay, but no more than five times. Prolonged culture *in vitro* or passaging can lead to reduced tumourigenicity *in vivo*.

### Preparation of cells to be injected • TIMING 30 min (day of injection)

- Harvest cells as required in a culture hood, and determine their concentration using a cell counter (either electronic or haematocytometer).
- Remove the appropriate amount of cell suspension required and centrifuge at 300 g for 3 min at room temperature.
  **★ CRITICAL STEP** You should always prepare approximately 20% extra cell suspension as some cells will be lost in the void volume of the needle and syringe.
- Discard the supernatant and resuspend the pellet in the appropriate volume of PBS (0.1 mL administered per mouse). Aliquot the cell suspension into eppendorfs and place on wet-ice to transport to the animal facility.

### Preparation of the recipient mice **• TIMING** 5 min (day of injection)

- In an appropriate procedure room, turn on the hotbox and allow it to reach the desired temperature (38 ± 1**°**C).
- Place mice inside the hotbox (each cage can go into a compartment of the hotbox, no need to separate the mice from one cage into individual compartments of the hotbox).
- Have the rodent restrainer in position, as well as the required number of syringes and needles (depending on the number of mice to be dosed).

### Injection of the mice **• TIMING** 10-40 min, depending on the number of mice

**★ CRITICAL STEP** You should be as efficient as possible, as the cells will deteriorate sitting in the PBS for long periods and you want to avoid a significant difference in the number of metastases between mice dosed at the beginning of a dosing session and the end of the session.

- Mix the gently cells (by inverting the tube several times), fill the syringe and place the needle on the end. **! CAUTION** Remove all air bubbles before placing the needle on the end of the syringe and then again be sure to push the plunger till the cell suspension comes out the end of the needle – air bubbles can lodge in the small capillaries of the mouse and cause immediate death.
- Immediately place a mouse from the hotbox into the restrainer and locate one of the two lateral tail veins (either by turning the tail 90**°** or rotating the whole restrainer 90**°**).
- Inject the tail vein with the desired volume (typically 0.1-0.2 mL) and then remove the mouse from the restrainer and place it back in its original cage as before the injection.
- Repeat steps 9-10 until the syringe is empty.
  **★ CRITICAL STEP** Ideally inject no more than ~5 mice per syringe to ensure that the cells are not sitting in the syringe for too long and have a chance to settle out of suspension. Use a fresh needle/syringe every ~5 mice to ensure the needle doesn’t become blunt (making it more painful for the mice) and no cells that have come out of suspension are mixed in with fresh cells suspension being drawn into the syringe. **! CAUTION** Never draw up cells into the syringe with the needle attached as it may lyse them.
- Monitor the mice in the cage for several minutes post-dosing to ensure there have been no ill effects from the procedure, before placing the cage back on the rack. For example, if too many cells are injected they may form an embolus and the mouse can die within a minute of dosing; typically the mice may start fitting or be very sluggish and reluctant to move, in which case they should be humanely sacrificed immediately and no further dosing at that cell concentration should be carried out.

### Determination of metastatic burden **• TIMING** 2 min to 2 days, depending on the number of mice and particular method used (3 methods listed below)

- Humanely sacrifice the mice as required by your local authority (it is preferable not to use an inhalation protocol, such as an anaesthetic compound or carbon dioxide, as this can induce lung pathologies, such as congestion and haemorrhaging, which make visualization of the metastases difficult).
- Remove the organ(s) of interest. Typically this will be the lung thus all steps hereafter only mention “lung”, however depending on the cell line used, this may also be the liver and/or other organs.
- Rinse the lung in PBS and dissect out all 5 lobes of the lung.
  **★ CRITICAL STEP** Be sure to collect (and count) all 5 lobes, as a single lobe will not necessarily be representative of the metastatic burden of the entire organ (since metastatic burden is never equally distributed between the lobes).

### Macroscopic counting of metastatic colonies **• TIMING** 2 min per mouse, depending on the number metastatic colonies (10-30 days post-injection, depending on the cell line used)

- Either by eye or with the use of a dissecting microscope, count the number of metastatic colonies on the surface of all 5 lobes of the lung, on both sides of the lungs to obtain the total metastatic burden for that mouse. **! CAUTION** Care needs to be taken if Bouin’s solution is used to fix and stain the lungs to aid visualization of the metastatic colonies as this reagent is toxic (thus needs to be done in a ventilated fume hood with gloves and a laboratory coat).

### Further analysis of metastatic burden

- Further analyses that can be undertaken are assessing H&E-stained paraffin sections (option A) or performing qPCR (option B).

(A) **H&E-stained paraffin sections** **• TIMING** **2 days for processing of the tissues and 5 min counting per mouse, depending on the number metastatic colonies**
  - Re-hydrate, paraffin-embed, section and stain with H&E using routine histopathology methods.
    PAUSEPOINT H&E-stained sections can be kept at room temperature indefinitely.
  - Count the number of metastatic colonies on the section of all 5 lobes of lung to obtain the total metastatic burden for that mouse.
(B) **qPCR** **• TIMING** **2 days for DNA extraction of the tissues and 1-2 hours for qPCR**
  - Homogenise all 5 lobes of the lung in 1 mL TBSTx (1x Tris-buffered saline with 0.5% Triton X-100).
  - Remove 100 uL of the homogenate and extract genomic DNA (we use Puregene Core A, Qiagen). The remainder of the homogenate can be stored at -20**°**C for future use if desired.
    PAUSEPOINT The extracted DNA can be kept at -20°C indefinitely.
  - Perform qPCR to determine the relative tumour burden – this can be achieved in a duplex reaction using a probe against a marker expressed in the tumour cells and a probe against a house-keeping gene (we use a FAM-conjugated mCherry probe and VIC-conjugated Vimentin probe that is primer limited as an internal control). Setup the qPCR according to the manufacturer’s instructions (we use Quantinova probe PCR kit) and perform 50 cycles of amplification.
    **★ CRITICAL STEP** The qPCR reaction should be performed in triplicate per tissue sample loading 50 ng of genomic DNA or 1 ng genomic DNA from the tumour cell line (positive control) or water and undosed tissue (both negative controls).
  - Once complete determine the cycle threshold for each well for both probes.
  - Derive the relative tumour burden using the following equation (using mCherry and Vimentin probes as examples):
    - Relative tumour burden = 100000 × 2^-ΔCT^
    - ΔCT = CT mCherry – CT Vimentin (where there is no mCherry amplification, the CT value is set to 50 or the number of cycles used for amplification).

## Timing

Step 1, passaging of the cells to be injected: 30 min (2-3 d before animal injection)

Step 2, preparation of cells to be injected: 30 min (day of injection)

Step 5, preparation of recipient mice for dosing: 5 min (day of injection)

Step 8, injection of the mice: 10-40 min (depending on the number of mice to be dosed)

Step 13, determination of metastatic burden: variable (depending on method used)

## ? Trouble Shooting

Trouble shooting advice can be found in Table 1.

## Anticipated Results

The anticipated results with the experimental metastasis assay depend on several factors. The number (and tissue site) of metastatic colonies formed will be dependent upon the cell line used to dose the mice, the dose of cells used, and the mouse genetic background, sex, age and mutant status. For large-scale mouse phenotyping of metastatic colonisation using a dose of 4×10^5^ mouse melanoma B16-F10 cells, wildtype C57BL/6 mice typically show 100 or 150 pulmonary metastatic colonies at 10 days post-dosing when analysed by macroscopic counts (for males and females, respectively; values ≤ 35 counts indicate the entire dose was not successfully administered into the vein). The successful procedure is dependent upon accuracy in locating and administering the full dosing volume to the tail vein, in a rapid and reliable manner.

## Figures and Tables

**Table 1:** Troubleshooting table.

Step	Problem	Possible reason	Solution
8	Difficulty locating the lateral tail vein	Mice are not sufficiently warm	Place mice back in the heated rodent chamber for a few minutes
8	Resistance when injecting the cell suspension into the tail vein	Needle not in the vein (in the peri-vascular tissue)	Remove needle and re-attempt the administration at a site further up the tail (towards the body of the mouse) or on the opposite lateral vein
11	No colonies present	The cell line was not metastatic	The cell line may have lost its *in vivo* metastatic properties through prolonged *in vitro* culture and/or drug selection. Thaw a fresh vial of cells and repeat experiment with as minimal passaging of the cells prior to use *in vivo* Repeat the experiment using a higher dose of cells
	Very few colonies present	Too few cells were used to dose the mice	Repeat the experiment using a higher dose of cells
11	Colonies too close together to be able to distinguish	Too many cells were used to dose the mice	Repeat the experiment using a lower dose of cells
11	Highly variable results	Mice are on a mixed genetic background	Increase the sample number
		Time taken to administer the number of doses in the syringe	Decrease the number of doses in each syringe so that the cells are not present in the syringe long enough for them to settle out of suspension
11	Significant difference in counts from the mice dosed at the start and end of the cohort	Time taken to dose the entire cohort was too long	Decrease cohort size or increase proficiency at dosing
14	Unable to clearly visualize colonies on the sections	Too much mountant or air bubbles within the mountant used to attach the coverslip to the slide	Increase proficiency at slide mounting or use automated mounting machine
		The tissue sections are too thick or ruffled or folded or show other sectioning artifacts	Cut thinner sections from the block or increase proficiency at operating microtome with water bath and cutting sections
14	Unable to distinguish metastatic colonies from other cell types	Significant immune infiltrate present in the tissue	Consider the use of immunohistochemistry using a tumour cell-specific biomarker antibody or a proliferative biomarker (e.g. Ki67) antibody to specifically identify the tumour cells and aid in distinguishing them from other cell types (e.g. such as macrophages or other immune cells)
		Cross-cutting through the edge of bronchial epithelium	Re-embed the lungs before re-sectioning, section deeper into the block, or seek the advice of an experienced pathologist
14	Too few colonies	Low dose was used or the mutant mouse may have a decreased metastasis phenotype	Repeat the experiment with a higher dose of cells or H&E stain multiple sections throughout the block of tissue to increase the area of tissue being examined
14	Colonies too close together to be able to distinguish	Too many cells were used to dose the mice	Repeat the experiment using a lower dose of cells
18	No signal on qPCR machine	Insufficient DNA was used or DNA was not sufficiently pure	Repeat the qPCR with the DNA sample. If vimentin probe is affected (this shows all DNA present), re-extract fresh DNA from the remainder of the frozen tissue lysate. If only the mCherry probe is affected, check cell line positive control to ensure the qPCR setup was correct.
18	Unexpected signal on qPCR machine	Unstable light sources from the instrument, or sample preparation problems (eg. bubbles in the reaction wells)	Check the amplification plots for quality of the fluorescence signal; the curves should be smooth during the exponential phase of the PCR. Any spikes in the curves indicate a problem. Check no bubbles are present in the wells and that the instrument is passing all its QC checks. Also check the seal of the wells in case some sample has evaporated during the analysis. If machine is from Applied Biosystems the ROX dye acts as an internal control.

## Experimental Design

An experimental metastasis assay in which cancer cells are injected into the tail vein of a mouse and the resulting secondary organ colonization assessed, primarily in the lung, 10 days later.
